# Insects Provide Unique Systems to Investigate How Early-Life Experience Alters the Brain and Behavior

**DOI:** 10.3389/fnbeh.2021.660464

**Published:** 2021-04-21

**Authors:** Rebecca R. Westwick, Clare C. Rittschof

**Affiliations:** Department of Entomology, University of Kentucky, Lexington, KY, United States

**Keywords:** critical period, phenotypic plasticity, genetic toolkit, trauma, DNA methylation

## Abstract

Early-life experiences have strong and long-lasting consequences for behavior in a surprising diversity of animals. Determining which environmental inputs cause behavioral change, how this information becomes neurobiologically encoded, and the functional consequences of these changes remain fundamental puzzles relevant to diverse fields from evolutionary biology to the health sciences. Here we explore how insects provide unique opportunities for comparative study of developmental behavioral plasticity. Insects have sophisticated behavior and cognitive abilities, and they are frequently studied in their natural environments, which provides an ecological and adaptive perspective that is often more limited in lab-based vertebrate models. A range of cues, from relatively simple cues like temperature to complex social information, influence insect behavior. This variety provides experimentally tractable opportunities to study diverse neural plasticity mechanisms. Insects also have a wide range of neurodevelopmental trajectories while sharing many developmental plasticity mechanisms with vertebrates. In addition, some insects retain only subsets of their juvenile neuronal population in adulthood, narrowing the targets for detailed study of cellular plasticity mechanisms. Insects and vertebrates share many of the same knowledge gaps pertaining to developmental behavioral plasticity. Combined with the extensive study of insect behavior under natural conditions and their experimental tractability, insect systems may be uniquely qualified to address some of the biggest unanswered questions in this field.

## Introduction

**FIGURE 1:**
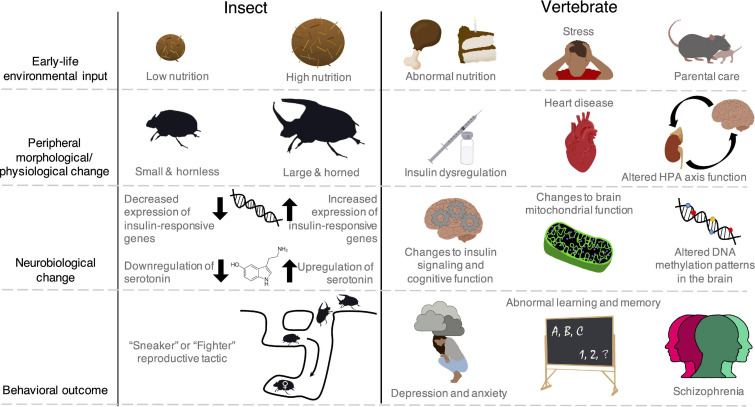
The impacts of early-life experiences extend beyond the brain to peripheral physiological systems and even body morphology in insect and vertebrate species. The brain and peripheral systems interact to shape adult behavioral expression in ways that remain poorly understood. Though these brain-peripheral connections are common across animals including vertebrates, and specifically humans, some insects show particularly conspicuous and discrete changes in morphology, presenting interesting systems to investigate behavioral regulation. Moreover, despite the more noticeable phenotypic differences in some insects, there are examples of common regulatory mechanisms (e.g., insulin signaling) that underpin behavioral dynamics across the insect and vertebrate phylogenetic space. Left: In some beetles (*Onthophagus spp.*), males that are provided high amounts of nutrition during development emerge as large adults with horns (Emlen, 1997). Horns give males a benefit in competition over female mates, which nest in sub-terranean tunnels under dung piles (Moczek and Emlen, 2000). These morphological changes are associated with changes in brain insulin and serotonin signaling (Snell-Rood and Moczek, 2012; Newsom et al., 2019) and result in two distinct male reproductive tactics. Large, horned males will guard female tunnels and compete with other rivals, while small, hornless males dig side tunnels and sneak around large males to reach the female (Emlen, 1997; Moczek and Emlen, 2000). Right: In vertebrates, early-life nutrition, stress, and social interactions cause coordinated changes in peripheral physiological function (Barker, 1995; Champagne and Curley, 2005; Avitsur et al., 2015) as well as brain hormone signaling, bioenergetics, and gene regulation (Hochberg et al., 2011; Korosi et al., 2012; Hoffmann and Spengler, 2018). These changes can give rise to cognitive and mental health disorders (Avishai-Eliner et al., 2002; van Os et al., 2010; Chen and Baram, 2016; Sripetchwandee et al., 2018).

Early-life experiences can have profound consequences for adult phenotypes, particularly behaviors (Beach and Jaynes, 1954), a phenomenon called developmental behavioral plasticity (sensu West-Eberhard, 2003, 2005). Although this phenomenon is well-established, its mechanistic basis remains a persistent research puzzle that touches many behavioral neuroscience disciplines and applications (Beldade et al., 2011; Snell-Rood, 2013; Reh et al., 2020). Brain development is fundamentally complex—it is a dynamic interaction between endogenous, gene-guided programs and environmental inputs (Boyce et al., 2020; Reh et al., 2020). Thus, determining how experiences are “embedded” requires knowledge at multiple levels of organization, from molecules to neural structure (Champagne, 2012; Cardoso et al., 2015; Curley and Champagne, 2016; Sinha et al., 2020). Moreover, individual differences can extend to peripheral tissues, which are also shaped by developmental experience and interact with the brain to influence adult behavioral expression (Figure 1). Finally, in addition to triggering behavioral change, environmental conditions dictate the adaptive consequences of behavioral expression. Understanding these consequences may allow researchers to predict the types of experiences that cause lasting or transient behavioral impacts. However, adaptive consequences of behavioral expression are difficult to ascertain in traditional lab-based model systems alone (Yartsev, 2017).

Fortunately, developmental behavioral plasticity occurs in animals as complex as humans and as simple as nematodes (Jobson et al., 2015; Kundakovic and Champagne, 2015). In this mini review, we explore how the insects are surprisingly well-suited to provide unique contributions to the study of this phenomenon. First, we highlight the strong ecological basis of insect behavior research (Schowalter, 2016), reviewing the exceptionally diverse systems available to explore the neurobiological basis of developmental behavioral plasticity in natural contexts with adaptive significance. Second, we provide an overview of the extensive examples of homology of function between insect and vertebrate nervous systems, despite their phylogenetic distance. We highlight the fact that a variety of mechanisms that embed developmental experience are broadly shared across groups. We conclude that insects offer a fertile and exciting area of future comparative research that explores the complex relationships between early-life experiences and adult behavioral expression.

## Insects as Models for Developmental Behavioral Plasticity in Natural Contexts

Extensive previous studies show that the developmental environment has diverse adaptive consequences for insect behavior. Such a perspective is valuable to behavioral neuroscience because environmental context defines the cues, sensory systems, and central processing dynamics that underpin behavioral change. Knowledge of environmental context may also be useful in establishing a general understanding of the types of conditions that give rise to transient versus lasting behavioral effects, a long-term goal in behavioral neuroscience. We highlight some of the established relationships between developmental experience and adult behavioral variation in insects, focusing on three major types of common environmental inputs: season, feeding experience, and interactions with other organisms.

### Season

Many insects integrate seasonal cues during development and adaptively tune their adult behavioral expression to match environmental conditions (De Wilde, 1962; Benoit, 2010; Buckley et al., 2012). For example, in the butterfly *Bicyclus anynana*, males produce a costly nutritional gift they provide to females in order to improve their mating chances. The costs and benefits of this gift change from the wet to the dry season, and accordingly, males adjust their gift production and courtship efforts depending on developmental moisture conditions (Prudic et al., 2011). In ground crickets (*Allonemobius fasciatus*), developmental temperature constrains male singing ability (Olvido and Mousseau, 1995), and as a result, females adjust their species-specific song preferences in response to their experience of temperature and day length during development (Grace and Shaw, 2004). Subtle differences in developmental temperature (e.g., developing in shaded versus sun-exposed shallow underground nests) can have profound behavioral impacts in female *Lasioglossum baleicum* bees; they shift from a cooperative reproductive tactic to a solitary one when developing in shadier locations (Hirata and Higashi, 2008). This selection of examples shows that the insects provide opportunities to investigate how simple developmental cues like temperature impact sophisticated phenotypes involving high level sensory integration and complex behaviors.

### Feeding Experience

Developmental feeding conditions can convey a variety of information. For example, because many insects are short-lived, developmental diet often predicts the state of nutritional resources available to the adult insect and even its offspring. Females of many insects, particularly moths, prefer to lay eggs on the same species of plant they fed on during development (Petit et al., 2015), a phenomenon often referred to as Hopkins’ Host Selection Principle (Hopkins, 1917). This pattern may minimize search time for suitable host plants for offspring. Though the mechanistic basis of this phenomenon remains controversial, experience-based developmental preferences for or against certain host plants or olfactory cues have been shown in multiple insect clades (Barron, 2001; Rietdorf and Steidle, 2002; Akhtar and Isman, 2003; Blackiston et al., 2008; Akhtar et al., 2009; Videla et al., 2010; Anderson et al., 2013; Anderson and Anton, 2014; König et al., 2015; Lhomme et al., 2017). Developmental feeding conditions can also indirectly signal the degree of intraspecific competition in the immediate environment, triggering mechanisms that alter myriad traits including adult body size, dispersal strategy, activity level, and exploratory behavior (Figure 1; Moczek and Emlen, 2000; Tripet et al., 2002; Tremmel and Müller, 2012).

Diverse neurobiological mechanisms are implicated in the response to developmental feeding experience. For example, plant volatile cues and the olfactory system play a strong role in butterfly and moth larval host plant identification (Petit et al., 2015). In other cases, including in some beetles, bees, aphids, and planthoppers, food intake itself is a cue leading to altered insulin and hormone signaling, which coordinate both peripheral and cognitive processes during development and throughout adulthood (Ament et al., 2008; Snell-Rood and Moczek, 2012; Zhang et al., 2019). More work is needed to understand how physiological processes like insulin signaling affect sensory perception and integration throughout adulthood, a topic that is currently of general interest in vertebrate cognitive neuroscience (Arvanitakis et al., 2020).

### Interactions With Other Organisms

Other animals (but see also Schretter et al., 2018; Schwab et al., 2018 for the role of microbiota) commonly shape the insect developmental environment. For example, in a variety of insects, conspecific density and predation pressure induce developmental behavioral plasticity (Walzer and Schausberger, 2011; Müller et al., 2016). One famous case involves the transition from the solitary to gregarious phase in migratory locusts. Increased frequency of physical contact during early life (a result of high conspecific density) gives rise to diverse morphological and behavioral changes, culminating in massive swarming events that disperse individuals to new locations with greater resources (Gillett, 1973; Simpson et al., 2001).

A variety of insect species (e.g., many ants, bees, wasps, and termites) live in complex eusocial societies where certain members forego reproduction to help raise the offspring of their relatives (Oster and Wilson, 1978). Individuals of these species interact socially with conspecifics throughout life, including during development. Female caste differentiation, where females can develop into either a reproductive queen or a non-reproductive worker, is a well-studied example of developmental behavioral plasticity in these eusocial insects (Schwander et al., 2010). Queen/worker caste determination is typically a function of larval nutrition (at least in part) and mediated by adult “nurses” who provide food to larvae (Brian, 1956; Gadagkar et al., 1991; Page and Peng, 2001; Liu et al., 2005; Smith et al., 2008). In some eusocial insects, particularly ants, developmental dietary differences also give rise to behaviorally and morphologically distinct “soldiers” (female workers specialized for defense; Rajakumar et al., 2018).

There are other more subtle effects of the developmental social environment in eusocial insects (Miura, 2004; Traynor et al., 2014; Wang et al., 2014). For example, worker honey bees express different levels of defensiveness during adulthood depending on the defensiveness of the nestmates who rear them; this effect may be mediated by diet, but it is subtle enough that it does not alter body morphology (Rittschof et al., 2015). Adult wasps use vibratory signals directed at larvae, in combination with dietary interventions, to influence adult behavior, again without conspicuous changes in morphology (Jandt et al., 2017). More primitive social insects also show effects of developmental social interactions. For example, in the twig-nesting small carpenter bee (*Ceratina calcarata*), a mother’s removal from the nest during the larval stage eliminates maternal grooming activity and increases defensive and avoidant behaviors once offspring reach adulthood (Arsenault et al., 2018). Behavioral differentiation in developing insects involves a variety of cue types (e.g., nutrition, pheromone, vibratory, or tactile signals), often acting in combination, suggesting that diverse sensory and physiological systems are integrated to give rise to behavioral effects.

## Homology in Insect and Vertebrate Nervous System Function and Plasticity

Insects have a popular reputation of having simplistic, decentralized nervous systems (Schaefer and Ritzmann, 2001). While it is true that some processes are locally guided by “ganglia,” semi-autonomous central nervous system components along the ventral nerve cord (Klowden, 2013), the brain is still required for sensory integration, decision-making, navigation, and learning (Pringle, 1940; Reingold and Camhi, 1977; Zill, 1986; Wessnitzer and Webb, 2006). Indeed, insects are capable of an impressive array of cognitive abilities, such as numeracy and social learning, because of their integrative brains (Chittka and Geiger, 1995; Giurfa et al., 1996, 2001; Dyer, 1998; Crist, 2004; Coolen et al., 2005; Avarguès-Weber, 2012; Pahl et al., 2013; Alem et al., 2016).

Insect brain structure and function is well studied (Ito et al., 2014), giving a strong basis to evaluate mechanisms of developmental plasticity from a comparative perspective. Extensive previous studies illuminate examples of homology of function with vertebrate systems (Simons and Tibbetts, 2019). Below we briefly review these general similarities, and then we focus on the specific neural mechanisms that encode developmental experience, many of which are also shared.

### Homology of Function Between Insect and Vertebrate Brains

Insect and vertebrate central nervous systems have similar functions (Kinoshita and Homberg, 2017), and many general features are shared, although notably, the evolutionary origin of these similarities remains controversial (Farris, 2008; Holland et al., 2013). For example, many of the same chemicals act as neurohormones and neurotransmitters, and even in conserved behavioral and cognitive contexts (Bicker et al., 1988; Osborne, 1996; Wu and Brown, 2006; Byrne and Fieber, 2017). In both vertebrates and insects including honey bees, bumble bees, fruit flies, and crickets, dopamine is involved in learning, novelty, reward prediction, and locomotion (Barron et al., 2008; Cohn et al., 2015; Gadagkar et al., 2016; Perry et al., 2016; Hattori et al., 2017; Terao and Mizunami, 2017; Felsenberg et al., 2018; Sovik et al., 2018). Likewise, serotonin modulates appetite, sleep, learning, social behavior, and aggression across a similar range of insect examples (Vleugels et al., 2015; Rillich and Stevenson, 2018; Bubak et al., 2020). Even insect-specific hormones have clear functional analogs in vertebrates. Insect juvenile hormone and vertebrate thyroid hormone both act through type II nuclear receptors, and they show similar growth and developmental functions (Flatt et al., 2006; Charles et al., 2011). Octopamine is an insect-specific neurohormone that is analogous to norepinephrine, and both compounds control stress response, motivation, and aggression (Roeder, 2005; Prieto Peres and Valença, 2010; Alfonso et al., 2019).

Beyond neurochemicals, recent studies suggest extensive homology between insect and vertebrate brain genome dynamics and protein function. Genes responsible for brain developmental patterning are surprisingly conserved (Lichtneckert and Reichert, 2005; Tessmar-Raible et al., 2007; Reichert, 2009; Loesel, 2011; O’Connell, 2013), and there is even evidence for functional conservation of genes associated with complex behaviors like territorial aggression, foraging, and brood care (Toth and Robinson, 2007; Rittschof et al., 2014; Toth et al., 2014; Saul et al., 2019; Shpigler et al., 2019). Cell types in the brain show similarities in structure and function. Like vertebrate brains, insect brains contain neurons and various types of glia (Losada-Perez, 2018), and the metabolic relationships between these cell types are similar across groups (Rittschof and Schirmeier, 2017). Neural activity is well-known for its energetic demands (Peters et al., 2004; Niven and Laughlin, 2008), and insects and vertebrates share some neural adaptations to high energy need (Robertson et al., 2020) and increased cognitive demands; the latter even shows a similar developmental basis (Farris, 2008).

**FIGURE 2:**
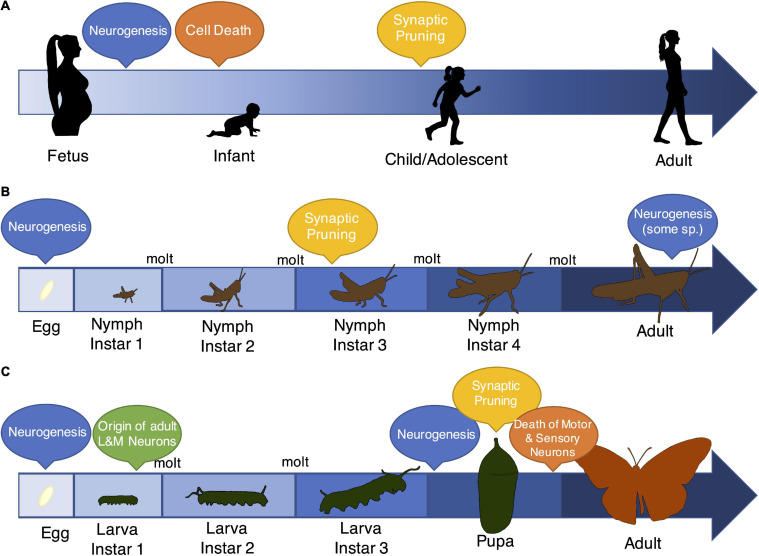
Patterns of development, specifically the timing of neurobiological events, vary across vertebrates and insects. Although insects and vertebrates show remarkable overlap in the types of mechanisms that characterize brain development and entrain early-life experience (Watson, 1992; Pearson, 1993; Reh and Cagan, 1994; Salzberg and Bellen, 1996; Luo and O’Leary, 2005; Bello et al., 2008), the progression of early-life, and specifically the timing of events like neurogenesis, programmed cell death (“Cell Death”), and synaptic pruning, differs markedly across these groups. **(A)** Most vertebrates show gradual changes in body size and tissue morphology. In the brain, they experience massive neurogenesis early in life followed by cell death and pruning through adolescence and early adulthood (Watson et al., 2006). Notably, more limited neurogenesis also occurs during adulthood (Zhao et al., 2008). **(B)** Some insects also show a pattern of gradual development (called “incomplete metamorphosis”), where juvenile stages resemble the basic body plan of adults. However, these insects still shed their exoskeletons in order to grow, and as a result, they transition through distinct developmental stages. Relatively little is known about neurobiological events in these species, although there is evidence of extensive neurogenesis both prior to egg hatch and during adulthood (Cayre et al., 1994). There is also evidence for synaptic pruning dynamics that resemble vertebrate mechanisms (Lnenicka and Murphey, 1989). **(C)** The majority of insects (∼80% of species) show a pattern of complete metamorphosis, where life stages have distinct morphologies, and adult behaviors and body plans vastly differ from juveniles. Data from several representatives of this group again suggest multiple periods of neurogenesis, both early in life and during the pupal stage (Booker and Truman, 1987; Truman and Bate, 1988). Interestingly, the timing of neurogenesis and programmed cell death and the retention of neurons through the life stages is brain region (and thus, functionally) specific (Wegerhoff, 1999; Tissot and Stocker, 2000). For example, a small number of neurons responsible for learning and memory originate early in the larval period and persist through adulthood, but most motor and sensory neurons are completely remodeled during the pupal phase (Cantera et al., 1994).

Despite extensive similarities, insects do show some profound differences in nervous system structure and function compared to vertebrates. For example, insect neurons are unmyelinated, they have different classes of olfactory and photoreceptors compared to vertebrates, and neuronal polarity is often different (Chittka and Niven, 2009; Kaupp, 2010; Gutierrez et al., 2011; Rolls and Jegla, 2015; Albert and Kozlov, 2016). Another conspicuous difference between insects and most vertebrates is the structure of early-life development (Figure 2), including the somewhat extreme behavioral and morphological changes that occur during insect metamorphosis. Metamorphosing amphibians and fish are notable exceptions within vertebrates and provide an exciting avenue for comparative work (Gilbert et al., 1996; Heyland and Moroz, 2006; Shi, 2013; Lowe et al., 2021). As with outward appearance, the structure and function of the nervous system can change dramatically during metamorphic developmental transitions in insects (Wolbert and Kubbies, 1983; Weeks and Truman, 1986; Gilbert et al., 1996). For instance, butterflies transition from relatively sessile plant-eating caterpillars to flighted adults with distinct diets, behavioral traits, sensory structures, and motor and cognitive capabilities (André, 1991; Ebenman, 1992). About 80% of all insect species (including ants, bees, wasps, butterflies, beetles, and flies, among others) experience this extreme form of metamorphosis (“complete metamorphosis,” Rolff et al., 2019). Most other insects experience incomplete metamorphosis, where the pupal stage is absent and the body plan in early life is more similar to that of the adult form (except for the absence of wings). Notably, some of these species still show radical differences in life history between juvenile and adult stages (Corbet, 1957; Gabbutt, 1959). The variation in development patterns in insects make them exciting but perhaps challenging subjects for comparative study of developmental behavioral plasticity.

Despite their developmental complexities, one unique benefit to insect study is that in some species, particularly those that undergo complete metamorphosis, only a subset of neurons is retained between the juvenile and adult stages (Figure 2; Cantera et al., 1994; Wegerhoff, 1999; Tissot and Stocker, 2000). This feature narrows the target populations for studies of early-life environmental effects. For example, in the sensory integration and learning and memory centers of the brain (primarily the “mushroom bodies”), adult neurons typically originate during early larval life, suggesting adequate opportunity to retain environmental information into adulthood; this is in contrast to sensory neurons, which are completely distinct between the larval and adult stages (Cayre et al., 1994; Tissot and Stocker, 2000). Moreover, even though the degree of neuronal remodeling may be relatively extreme in insects compared to vertebrates, the components of the remodeling process closely resemble the types of developmental changes that also occur in vertebrates (Luo and O’Leary, 2005; Bello et al., 2008). For example, analogous to developing vertebrates, different neuron populations in circuits associated with learning and memory display a coordinated process of pruning and regrowth during metamorphosis in *Drosophila melanogaster* (Spear, 2013; Mayseless et al., 2018). These features of insect neurodevelopment provide unique opportunities to study the complex neural mechanisms of developmental behavioral plasticity in careful detail.

### Homology of Function in Neural Mechanisms that Encode Developmental Experience

Early-life cues change adult behavior by persistently altering the structure and/or function of the nervous system (Odeon et al., 2013). Though the precise dynamics of these changes remain poorly understood in any system, in general terms, known mechanisms are similar when comparing vertebrates to insects (Watson, 1992; Pearson, 1993; Reh and Cagan, 1994; Salzberg and Bellen, 1996). Major categories of mechanisms include epigenetic modifications, changes in the quantity of neurochemicals and/or their receptors, and brain structural changes (Elekonich and Robinson, 2000; Kretzschmar and Pflugfelder, 2002; Fahrbach, 2006; Schoofs et al., 2017; Glastad et al., 2019). These mechanisms are not mutually exclusive, and one long-term challenge in behavioral neuroscience for insects and vertebrates alike is to understand how these mechanisms are integrated to alter dynamic behaviors (Wolf and Linden, 2011). However, here we highlight some known insect examples of epigenetic, neurochemical, and structural mechanisms that encode developmental experience.

Chemical modifications to brain DNA are proposed to be critical mediators of early-life effects on adult behavior in vertebrates (Aristizabal et al., 2019). DNA methylation and histone post-translational modifications are the most well-studied among these mechanisms (Smallwood and Kelsey, 2012; Paredes et al., 2016). Not all insects possess appreciable levels of DNA methylation (Deobagkar, 2018; Deshmukh et al., 2018), but some, including many social insects, do (Li-Byarlay, 2016; Yagound et al., 2020). Some studies show that developmental experience-induced changes in DNA methylation impact adult behavioral phenotypes (Linksvayer et al., 2012; Patalano et al., 2012; Weiner and Toth, 2012; Yan et al., 2014; Alvarado et al., 2015). For example, the variation in larval diet that gives rise to queen versus worker female honey bees acts at least in part through DNA methylation changes in both the head and peripheral tissues (Kucharski et al., 2008; Shi et al., 2011; Wang et al., 2020). Similarly, studies in termites and locusts demonstrate a relationship between differential DNA methylation and developmentally induced adult behavioral variation (e.g., in the solitary versus gregarious phases of migratory locusts, Lo et al., 2018). Other known epigenetic mechanisms also play a role in developmental behavioral plasticity in insects, including histone modifications and long non-coding RNAs (Simola et al., 2016; Glastad et al., 2019).

The relationship between brain epigenetic modifications and gene expression patterns varies across species and is not well-understood. For example, whereas DNA methylation in gene regulatory regions tends to suppress gene expression in vertebrates, in insects, gene body methylation, which is thought to regulate alternative splicing, is more common (Feng et al., 2010; Zemach et al., 2010; Glastad et al., 2014; Schmitz et al., 2019). Furthermore, some studies have shown surprisingly weak relationships between DNA methylation dynamics and behavioral expression (Herb et al., 2012; Libbrecht et al., 2016). More data is necessary to understand how DNA methylation dynamics correspond to both gene expression dynamics and behavior (Flores et al., 2012; Li-Byarlay, 2016; Jeong et al., 2018), including whether the presence and degree of DNA methylation and other epigenetic modifications predict capacity for behavioral plasticity (Kapheim et al., 2015; Lo et al., 2018). These are general challenges facing vertebrate research as well (Di Sante et al., 2018), which could benefit from a comparative approach.

The developmental environment can cause lasting behavioral effects by altering neurochemical processes, e.g., circulating levels of hormones and neurotransmitters in the central nervous system. For example, changes in brain insulin, juvenile hormone, prothoracicotropic hormone, octopamine, and serotonin signaling are prominent correlates of insect developmental behavioral plasticity (De Wilde and Beetsma, 1982; Rachinsky, 1994; Paulino Simões et al., 1997; Moczek and Emlen, 2000; Snell-Rood and Moczek, 2012; Erion and Sehgal, 2013; Newsom et al., 2019). These chemicals impact behaviors like aggression, gregariousness, feeding, locomotion, and non-aggressive social interactions (Iba et al., 1995; Anstey et al., 2009; Erion and Sehgal, 2013) in a number of species, including the cricket and locust examples above. The degree to which neurochemical systems comparably regulate behaviors across vertebrates and invertebrates is a matter of debate (Bubak et al., 2020), and thus an important area of on-going study, especially in the context of developmental behavioral plasticity.

A final common way the developmental environment affects the nervous system is through brain structural changes (Teicher et al., 2016; Saleh et al., 2017; Hall and Tropepe, 2020). For example, in flies, high conspecific density during development results in larger mushroom bodies and enhanced olfactory processing abilities (Heisenberg et al., 1995). Similar conditions in wasps lead to increased overall adult brain size, and larger-volume mushroom bodies and regions required for visual processing (Groothuis and Smid, 2017). Gregarious locusts have larger integrative mushroom bodies, while solitary individuals show neural adaptations associated with enhanced sensory sensitivity (Ott and Rogers, 2010). Female social insects often show variation in relative brain region size as a function of behavioral specialization (Lucht-Bertram, 1961; Wheeler and Nijhout, 1984; Vitt and Hartfelder, 1998; Page and Peng, 2001; Muscedere and Traniello, 2012). Insect and vertebrate nervous systems not only exhibit many of the same developmental plasticity mechanisms, but they also face many of the same conceptual challenges associated with connecting developmental experience to behavioral expression. These extensive similarities suggest many potential benefits to comparative study.

## Discussion

Predicting, and in some cases changing, adult behavioral effects of early-life experience are challenges relevant to diverse fields of behavioral neuroscience (West-Eberhard, 2003; Beldade et al., 2011; Bryck and Fisher, 2012; Snell-Rood, 2013; Stamps and Biro, 2016; Danese, 2020; Reh et al., 2020). Behavioral effects of early-life experience are commonplace among animal species, presenting the opportunity to use comparative approaches to identify the general principles of developmental behavioral plasticity. Many fundamental questions that are common to both insects and vertebrates remain to be resolved, for example, how the brain integrates early-life experience across multiple levels of organization, and whether specific mechanisms like DNA methylation universally predict long term behavioral impacts. Moreover, it remains unclear how developmental experiences are integrated with other sources of information (e.g., genetic variation, parental transgenerational effects) that also influence behavior (Dall et al., 2015; Stamps and Frankenhuis, 2016; Stein et al., 2018; Rösvik et al., 2020), and whether these outcomes can be modified by additional information later in life. Though these sources of complexity apply to both insect and vertebrate species, certain characteristics of insects, like their relatively short lifespans, may alter the ecological selection pressures that shape information integration. With respect to the evolution and expression of behavioral plasticity, diverse comparative approaches may illuminate both broad, general features and taxon-specific patterns.

In insects, studies of behavioral plasticity largely focus on processes during the adult stage, and although many patterns of nervous system development are known (Prillinger, 1981; Rospars, 1988; Hähnlein and Bicker, 1997; Cayre et al., 2000; Awasaki et al., 2008), precisely how these patterns respond to early-life environmental stimuli remains poorly understood. However, the deep research history of insects in natural ecological contexts provides diverse, tractable systems for future work that fills this research gap. The developmental environment, including simple abiotic factors like temperature and moisture, impacts a variety of sophisticated behaviors from dispersal patterns (Zera and Denno, 1997; Alyokhin and Ferro, 1999; Benard and McCauley, 2008) to social and reproductive tactics (Radwan, 1995; Emlen, 1997; Taborsky and Brockmann, 2010; Łukasik, 2010; Kasumovic and Brooks, 2011). Thus, in controlled but environmentally relevant experiments, it is possible to assess how specific types of developmental inputs shape both sensory and integrative processes (Anton and Rossler, 2020; Fernandez et al., 2020; Gonzalez-Tokman et al., 2020) relevant to many different behavioral phenotypes. In addition, the short generation time of insects is ideal for life-long studies of behavior.

On the neurobiological level, developmental behavioral plasticity in insects is mediated through familiar neural plasticity mechanisms like epigenetic modifications, neurochemical changes, and changes to neural structure (LeBoeuf et al., 2013). Some of these mechanisms can be, and have been, explored in the context of traditional learning and memory frameworks, which also are well established in insects (Tully et al., 1994; Blackiston et al., 2008; Yang et al., 2012; Alloway, 2015; Tan et al., 2015). Though most learning and memory research has focused on dynamics during the adult stage (Fahrbach et al., 1998; Ravary et al., 2007; Li et al., 2017; Jernigan et al., 2020), many insights from this work are likely applicable to the pre-adult life stages as well. Moreover, in what may be the majority of insect species, only a subset of the brain survives the transition from the juvenile life-stage to adulthood, presenting a narrow range of target areas in which to carefully investigate how neural plasticity mechanisms give rise to complex behaviors. However, some challenges to comparative work remain. For instance, it is unclear which insect life stages are comparable to the early-life timeframe in vertebrates, or whether retention of early-life effects in insects is fundamentally constrained by their extensive morphological and neurobiological remodeling (Vea and Minakuchi, 2020).

Despite these challenges, insects have a history of contributing surprisingly general insights into complex behavioral phenotypes relevant to vertebrate species. For example, eusocial insects present detailed systems to address general neurobiological principles of developmental behavioral plasticity in the context of complex social living. Because insect societies show patterns of organization that can be generalized to other social species (Seeley, 1995; Bonner, 2004; Ireland and Garnier, 2018), they have tremendous promise for investigating both the causes and consequences of developmental plasticity in vertebrates. This comparison may even extend to humans, where many persistent effects of the early-life environment on behavior and mental health are social in nature (Miller et al., 2009; Nothling et al., 2019). It is possible that behavioral plasticity in social contexts has unique neurobiological features (Taborsky and Oliveira, 2012), and social insects will continue to serve as excellent models to examine this idea.

Although this review is specifically focused on insect contributions to behavioral neuroscience in a comparative framework, the uniqueness of this animal group, as well as its ecological and economic importance, cannot be overstated. These aspects provide further motivation for study of developmental behavioral plasticity in this group. Many bee species are important agricultural pollinators (Winfree et al., 2011; Reilly et al., 2020). The ongoing locust outbreak in East Africa is anticipated to cause enormous economic loss and endanger food security (Peng et al., 2020). Many agricultural pests are metamorphosing insects with destructive larval feeding stages (e.g., beetles and moths). Understanding the natural history of these organisms, as well as the range of neural and behavioral responses to developmental experience (Haynes, 1988; Desneux et al., 2007; De França et al., 2017; Müller, 2018; Sehonova et al., 2018) will improve environmental management in addition to deepening our understanding of the general principles of developmental behavioral plasticity.

## Author Contributions

RW and CR conceptualized and wrote the manuscript. Both authors contributed to the article and approved the submitted version.

## Conflict of Interest

The authors declare that the research was conducted in the absence of any commercial or financial relationships that could be construed as a potential conflict of interest.
